# Anaemia and Its Relation to Demographic, Socio-economic and Anthropometric Factors in Rural Primary School Children in Hai Phong City, Vietnam

**DOI:** 10.3390/nu11071478

**Published:** 2019-06-28

**Authors:** Ngan T.D. Hoang, Liliana Orellana, Tuyen D. Le, Rosalind S. Gibson, Anthony Worsley, Andrew J. Sinclair, Nghien T.T. Hoang, Ewa A. Szymlek-Gay

**Affiliations:** 1 Institute for Physical Activity and Nutrition (IPAN), School of Exercise and Nutrition Sciences, Deakin University, Melbourne, VIC 3125, Australia; 2 Biostatistics Unit, Faculty of Health, Deakin University, Melbourne, VIC 3125, Australia; 3 National Institute of Nutrition, Hanoi 10000, Vietnam; 4 Department of Human Nutrition, University of Otago, PO Box 56, Dunedin 9054, New Zealand; 5 Faculty of Health, Deakin University, Geelong, VIC 3216, Australia; 6 Department of Nutrition, Dietetics and Food, Monash University, Clayton, VIC 3168, Australia; 7 Nutrition Society of Australia, PO Box 576, Crows Nest, NSW 1585, Australia; 8 Hanoi Medical University, Hanoi 10000, Vietnam

**Keywords:** Vietnam, anaemia, childhood overweight/obesity, malnutrition, double burden of diseases, school children

## Abstract

Little is known about the prevalence of anaemia and associated factors in school children in Vietnam. In this cross-sectional study, we aimed to determine the prevalence of anaemia and its subtypes, and the associations of types of anaemia with demographic, socio-economic and anthropometric factors among 6–9-year-old primary school children in rural areas of Hai Phong City, Vietnam. Haemoglobin (Hb) and mean corpuscular volume (MCV) were measured, and demographic, socio-economic and anthropometric data were collected in 893 children from eight primary schools. The prevalence of anaemia (Hb < 115 g/L) was 12.9% (95% CI: 8.1%, 19.9%), microcytic anaemia (Hb < 115 g/L and MCV < 80 fL) was 7.9% (95% CI: 5.3%, 11.6%) and normocytic anaemia (Hb < 115 g/L and MCV 80–90 fL) was 5.3% (95% CI: 2.9%, 9.5%). No child presented with macrocytic anaemia (Hb < 115 g/L and MCV > 90 fL). Children who were underweight, wasted, or in anthropometric failure (either underweight, stunted or wasted) were more likely to be anaemic (all *p* ≤ 0.004), and specifically, to have normocytic anaemia (all *p* ≤ 0.006), than those who were not underweight, wasted or in anthropometric failure. Stunted children were more likely to be anaemic (*p* = 0.018) than those who were not stunted. Overweight/obese children were less likely to be anaemic (*p* = 0.026) or have normocytic anaemia (*p* = 0.038) compared with children who were not overweight/obese. No anthropometric status indicator was associated with the risk of microcytic anaemia. No demographic or socio-economic factor was associated with any type of anaemia. Anaemia remains a public health issue in rural areas in Hai Phong City, Vietnam, and future approaches for its prevention and control should target undernourished primary school children.

## 1. Introduction

Anaemia is used as an indicator of nutritional and health status in children [1] because of its adverse effects on childhood morbidity and mortality. Anaemia reportedly causes 600,000 child deaths in low- and middle-income countries annually [2], with the highest proportion of deaths occurring in South Asia. It is not only a risk factor for impaired physical activity performance and school participation in children, but it may also persist into adulthood. Anaemia in adulthood may lead to negative economic consequences because it has been shown to result in low productivity due to reduced work capacity [3,4,5]. Chronic inflammatory disorders, parasite infections, malaria [6], genetic haemoglobin disorders and co-existing micronutrient deficiencies of iron, folic acid, vitamin B12, and vitamin A [1,7] play a role in the occurrence of anaemia. Of these, iron deficiency is often assumed to be a major cause of anaemia [8]. Lower age [9], male sex [10], lower maternal education [11], and lower household income [12] have been associated with increased risk of anaemia in childhood. Therefore, effective strategies to prevent and control anaemia must not only rely on understanding the causes of the condition, but must also determine how these may affect vulnerable population groups.

Children in low-income countries are particularly at risk of anaemia because their intake of readily bioavailable haem iron from flesh foods is often inadequate in relation to their requirements for growth. Additional exacerbating factors may include malabsorption from gastrointestinal diseases and excessive iron losses (for example during hookworm infection, or malaria) [13]. Undernutrition has also been shown to be associated with anaemia in children [14].

Recently, children with greater adiposity have been shown to be at risk of anaemia because of their higher risk of micronutrient deficiencies such as iron, vitamin A, or zinc [15], and increased hepcidin concentration due to chronic inflammation in overweight/obese children, leading to decreased iron absorption [16]. While iron deficiency can be attributed to approximately half of all anaemia cases [17], it is more frequent in overweight/obese children compared with normal weight children [16,18,19,20]. To date, limited research has investigated the association between anaemia and childhood overweight/obesity. Studies in overweight/obese children have mainly concentrated on iron deficiency, a single cause of anaemia [16,18,19,20].

In Vietnam, the prevalence of anaemia among school children in rural areas ranged from 25% [21] to 45% a decade ago [22], but current data for the prevalence and associated factors of anaemia in this age group are still limited in some provinces [21,22,23,24], and there is a lack of data for the prevalence of anaemia in overweight and obese children. This lack of data in school children may be due to the fact that during the last 30 years, nutrition policies have prioritised malnutrition prevention in children under five years of age [25]. Therefore, the majority of studies have investigated the associations of anaemia and socio-economic conditions in preschool children, which created a gap for anaemia research in school-age children. Thus, an investigation of the prevalence of anaemia in school-age children and its relationship with demographic, socio-economic and anthropometric status indicators is required.

The aims of this study were (1) to estimate the prevalence of anaemia and its subtypes among 6–9-year-old primary school children, in rural areas of Hai Phong City, Vietnam; and (2) to explore associations of anaemia and its subtypes with demographic and socio-economic factors and anthropometric status in order to identify at-risk children.

## 2. Materials and Methods

### 2.1. Study Design

This study used screening data from a randomised controlled trial that investigated the effects of micronutrient supplementation for improving micronutrient status, and growth, health and cognitive outcomes in primary school children in Vietnam (registered at www.actr.org.au as ACTRN12616001245482). This cross-sectional study is based on blood samples and anthropometric measurements collected in October 2016 from 6–9-year-old children (*n* = 893) attending eight primary schools in rural areas in Hai Phong City, Vietnam. We also collected self-reported socio-economic data from the children’s mothers. The Ethics Committee of the National Institute of Nutrition, Vietnam (610/VDD-QLKH) and the Deakin University Human Research Ethics Committee, Australia (2016-181) approved the study protocol. Written informed consent was obtained from a primary caregiver of each child taking part in the study, and verbal consent was obtained from each participating child.

### 2.2. School Selection

Hai Phong City is administratively divided into eight rural and seven urban districts; this research was conducted in rural districts only. A multi-stage sampling approach was used to select eight schools as described in detail elsewhere [26]. Briefly, two districts were chosen at random from the eight rural districts in Hai Phong City in the first stage, followed by the selection of eight schools from all primary schools in each district in the second stage.

### 2.3. Participant Recruitment

All children attending grades 1–3 (aged 6–9 years) in the participating schools were invited to take part. The child’s date of birth was taken from the school registration form.

Children were excluded if they were older than 108 months, had a diagnosed chronic haematological disease confirmed by the parents (e.g. chronic anaemia resulting from long-term health conditions such as cancers, auto-immune disorders or long-term infections; thalassemia; or other diagnosed disease that would affect interpretation of haemoglobin data), had an anthropometric abnormality (e.g. severe scoliosis which would not allow for correct determination of height), or had an intellectual impairment that would prevent them from understanding the aims of this research.

### 2.4. Blood Sample Collection and Haematological Measures

Non-fasting venous blood was drawn into 2-mL EDTA evacuated tubes (Vacuette, Greiner Bio One, Kremsmünster, Austria) by trained staff. The tubes were immediately refrigerated at 4 °C and transferred to the Hai Phong Preventive Health Centre within 6 hours of sample collection where samples were analysed. Whole blood was used to determine haemoglobin (Hb) and mean corpuscular volume (MCV) with a Micros ES 60 automated haematology analyser (HORIBA ABX, France).

The Hb and MCV measurements were validated through participation in the Randox International Quality Assessment Scheme. A pooled blood sample and certified reference material (Randox Laboratories Limited, Crumlin, UK) were analysed monthly for Hb concentration and MCV to assess the precision and accuracy of the analytical methods. The analysed mean value for the pooled blood sample was 144.4 g/L (*n* = 5,503) for Hb, and 85.7 fL (*n* = 5,215) for MCV. The analysed mean value for the quality control certified reference material was 145.8 g/L for Hb, compared to the manufacturer’s reference range of 131.7–146.7 g/L, and 89.4 fL for MCV, compared to the manufacturer’s reference range of 76.3–90.3 fL. For Hb, the coefficient of variation was 2.1% for the pooled blood sample and 1.9% for the certified reference material. For MCV, the coefficient of variation was 5.0% for the pooled blood sample and 3.3% for the certified reference material. Additionally, 10% of the study samples were analysed in duplicate for Hb and MCV. The coefficients of variation for the duplicate samples were 1.7% for Hb and 2.8% for MCV.

Anaemia was defined as Hb < 115 g/L [27]. Anaemia was further classified as microcytic anaemia (i.e., anaemia of iron deficiency, chronic disease, and/or haemoglobinopathies; Hb < 115 g/L and MCV < 80 fL) [27,28], normocytic anaemia (i.e., anaemia of chronic inflammation; Hb < 115 g/L and MCV 80–90 fL) [27,28,29], and macrocytic anaemia (i.e., anaemia of folate deficiency or vitamin B12 deficiency; Hb < 115 g/L and MCV > 90 fL) [27,29].

### 2.5. Anthropometric Measures

All anthropometric measurements were taken twice from each child by trained staff from the National Institute of Nutrition according to standardised procedures [30]. Children were weighed to the nearest 0.1 kg in light clothing and without shoes, using calibrated electronic body scales (TANITA BC-543, TANITA Corporation, Tokyo, Japan). Height was measured to the nearest 0.1 cm with a SECA stadiometer (SECA 222, SECA GMBH & Co. KG, Hamburg, Germany). A third measurement was taken if the two measurements differed by more than 0.1 kg for weight and 0.1 cm for height. The final weight and height were calculated as the mean of the two or three measurements obtained for each child, as appropriate.

Body Mass Index (BMI) was calculated as weight in kilograms divided by the square of height in meters. Height-for-age, weight-for-age, and BMI-for-age z-scores were calculated with the WHO Anthro Plus software version 2.0 [31]. The WHO classification was used to define stunting (i.e., height-for-age z-score < −2), underweight (i.e., weight-for-age z-score < −2), wasting (i.e., BMI-for-age z-score < −2), and overweight/obesity (i.e., BMI-for-age z-score > 1) [32]. The Composite Index of Anthropometric Failure (CIAF), which is an aggregate indicator of malnutrition and provides a single measure of the severity of undernutrition in the population [33], was used to identify anthropometric failure. Children who presented with at least one of the indicators of undernutrition (i.e., stunting, underweight, or wasting) were classified to be in anthropometric failure [33].

### 2.6. Socio-economic Status Data

The children’s mothers provided information on their current employment status, education level and monthly household income via a self-administered questionnaire.

### 2.7. Data Analysis

In total, 3960 children were invited to participate in this study. Of these, 2334 children agreed to participate and provided anthropometric data and 893 of them also provided a blood sample and were included in the current analysis. All consenting children fulfilled the study eligibility criteria. Sex, age and Hb concentration were available for all 893 children. Weight-for-age z-score and CIAF were available for 890 children; height-for-age and BMI-for-age z-scores were available for 888 children. MCV was available for 860 children. Maternal employment status was reported by 673 (75.4%) mothers, maternal education by 678 (75.9%) mothers, and monthly household income by 640 (71.7%) mothers.

Children’s age was classified into tertiles (69 to < 81 months, 81 to < 92 months, and 92 to ≤ 108 months). High school education in Vietnam is comprised of 3 years of formal schooling, grades 10 to 12, and is normally the minimum qualification required to obtain employment. Therefore, maternal education was classified into three categories: above high school, high school, and below high school. Monthly household income was classified as < 6.0 million VND, 6.0 to < 10 million VND, and ≥ 10 million VND.

The demographic, socio-economic and anthropometric data were compared between participants included in this study and those who did not provide a blood sample (*n* = 1441) using linear mixed models (LMMs) for continuous outcomes (age, weight-for-age z-score, height-for-age z-score, BMI-for-age z-score, and monthly household income) and generalised LMMs for binary variables (sex (boy/girl), maternal employment status (full time employment and self-employed [indicating better income]/farmer and unemployed [indicating poorer income]), and maternal education (high school and above/below high school)) with participation as the fixed effect and school as a random effect.

Generalised LMMs were also fitted to estimate the prevalence of each type of anaemia, and to assess the associations between each type of anaemia and demographic and socio-economic factors (sex, age, maternal employment status, maternal education, and monthly household income), and anthropometric status indicators (stunting, underweight, wasting, overweight/obesity, and anthropometric failure). All models included school as a random effect to account for clustering. We report univariate associations between each type of anaemia and each demographic or socio-economic factor or anthropometric indicator. Even though no demographic variable was associated with any type of anaemia in our sample, we also fitted models adjusting for sex and age. As the results of these multivariate models were similar to the unadjusted models, we only report in tables the unadjusted results.

The intracluster correlation coefficient estimated for each type of anaemia under a model that included school as a random effect and no covariates is also reported. The coefficient measures the degree of heterogeneity for the prevalence of each type of anaemia across schools.

We also plotted Hb concentration against MCV to visualise the unadjusted prevalence of each type of anaemia and cases of no anaemia.

To facilitate comparisons with published data, we conducted additional analyses where we assessed associations between Hb concentration and BMI-for-age z-score and socio-economic factors. A LMM was fitted with Hb concentration as the outcome, BMI-for-age z-score as the fixed effect, and school as a random effect, while adjusting for sex and age. We also used LMMs to assess the association between Hb concentration and socio-economic factors, with all models including school as a random effect.

All estimates are reported along with 95% confidence intervals (CI). All analyses were performed with Stata (version 14.0; Stata Corp LP, College Station, Texas, USA).

## 3. Results

### 3.1. Participants

A similar proportion of girls and boys took part in the study (Table 1). In the whole sample, 5.3% of children were stunted, 8.8% were underweight, and 6.2% were wasted. Both anthropometric failure and overweight/obesity were common, affecting more than 1 in 8 children (13.3%) and 1 in 6 children (18.7%), respectively. The majority of children’s mothers were currently engaged in full time employment, and had an education level at or above high school.

Children included in this study (*n* = 893) had a lower weight-for-age z-score (difference of 0.10; 95% CI: −0.17, −0.03), height-for-age z-score (difference of 0.10; 95% CI: −0.19, −0.01), and BMI-for-age z-score (difference of 0.09; 95% CI: −0.15, −0.02), and their mothers were less likely to be farmers or unemployed (OR: 0.71; 95% CI: 0.56, 0.90), compared with children who did not provide a blood sample. Sex, age, maternal education, and monthly household income did not differ between these two groups of children.

### 3.2. The Prevalence of Anaemia and Associated Factors

Of the 893 participating children, 138 (15.5%) had anaemia. The estimated prevalence of anaemia, after accounting for clustering, was 12.9% (95% CI: 8.1%, 19.9%). Anaemia was not significantly associated with any of the demographic or socio-economic variables, but was significantly associated with all anthropometric status indicators in univariate analyses (Table 2). After adjusting for sex and age, all anthropometric status indicators remained significantly associated with anaemia with similar OR estimates (all *p* ≤ 0.022; results not shown in tables).

### 3.3. The Prevalence of Microcytic Anaemia and Associated Factors

Microcytic anaemia was present in 77 (9.0%) children. The estimated prevalence of microcytic anaemia, while accounting for clustering, was 7.9% (95% CI: 5.3%, 11.6%). No demographic or socio-economic factor or anthropometric status indicator was significantly associated with microcytic anaemia in univariate analyses (Table 3).

### 3.4. The Prevalence of Normocytic Anaemia and Associated Factors

Among the children who participated in the study, 59 (6.9%) had normocytic anaemia. After accounting for clustering, the prevalence of this anaemia was 5.3% (95% CI: 2.9%, 9.5%). In univariate models, normocytic anaemia was not associated with any demographic factors, socio-economic status, or stunting but it was significantly associated with underweight, wasting, anthropometric failure, and overweight/obesity (Table 4). After adjusting for sex and age, underweight, wasting, anthropometric failure, and overweight/obesity remained significantly associated with normocytic anaemia and the OR estimates were similar (all *p* ≤ 0.047; results not shown in tables). 

### 3.5. Relationship between Hb and MCV, BMI-For-Age Z-Score, and Socio-Economic Factors

**Figure 1:**
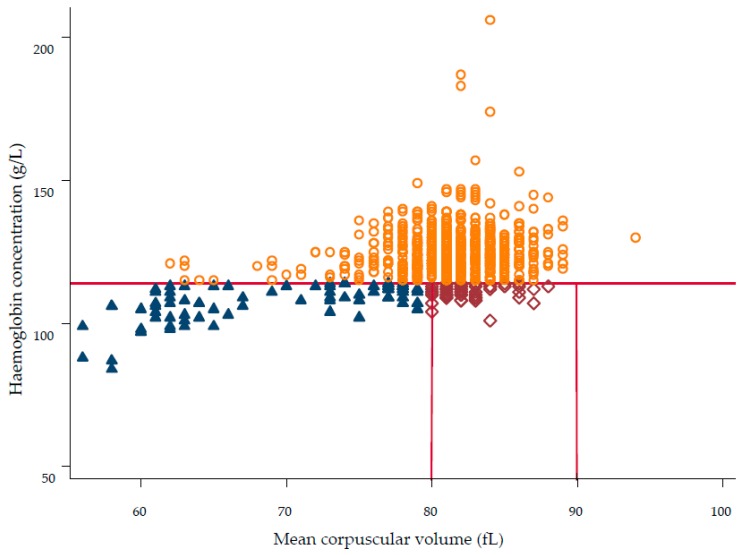
Scatter plot depicting the relationship between haemoglobin concentration and mean corpuscular volume in 6–9-year-old rural primary school children (*n* = 860) in eight primary schools in Hai Phong City, Vietnam. Microcytic anaemia was defined as Hb < 115 g/L and MCV < 80 fL (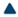, *n* = 77, 9.0%), normocytic anaemia as Hb < 115 g/L and MCV 80–90 fL (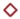, *n* = 59, 6.9%), macrocytic anaemia as Hb < 115 g/L and MCV > 90 fL (*n* = 0, 0.0%), and no anaemia was defined as Hb ≥ 115 g/L (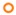, *n* = 724, 84.1 %).

Figure 1 shows the relationship between Hb concentration and MCV. Of the three types of anaemia, microcytic anaemia was most common with 77 of the 860 children affected. Anaemia of chronic inflammation (normocytic anaemia) was evident in 59 children, while no children in the sample presented with macrocytic anaemia.

Hb concentration was significantly associated with BMI-for-age z-score (β = 0.13; 95% CI: 0.09, 0.18) but showed no association with any of the socio-economic factors (all *p* ≥ 0.188).

### 3.6. Intracluster Correlation Coefficients

The estimated intracluster correlation coefficients for anaemia, microcytic anaemia and normocytic anaemia are reported in Table 5. This information may inform sample size calculations in future studies aiming to estimate the prevalence of each type of anaemia in school children in Vietnam.

## 4. Discussion

This cross-sectional study found that anaemia remains a public health issue in rural areas of Hai Phong City, with 12.9% of children estimated at risk. Microcytic anaemia appeared to be more common in this sample of children (7.9%) than normocytic anaemia (5.3%), with no child presenting with macrocytic anaemia. Underweight, wasting, and anthropometric failure were associated with higher odds of anaemia and normocytic anaemia, and stunting was associated with a higher risk of anaemia. Overweight and obese children were less likely to have anaemia or normocytic anaemia. No anthropometric status indicators were associated with the risk of microcytic anaemia. We also found no evidence of an association between anaemia or its subtypes and any of the selected demographic or socio-economic factors.

The prevalence of anaemia in this study was lower than the findings from earlier studies in Vietnam [21,34,35] which were 25.0% among 400 primary school children in rural areas in Phu Tho province in 2003 [34] and 23.6% among 1229 children aged 6–9 years in six primary schools in poor rural areas in Northern Vietnam in 2006 [35]. The lower prevalence of anaemia in rural school children noted in our study may be explained by Hai Phong City’s greater socio-economic development compared to other cities/provinces of Vietnam [36]. It is also possible that there has been a decrease over the last decade in the proportion of children affected by anaemia in Vietnam as our results are comparable to that of a large study conducted in 2011 in 2872 children aged 0.5–11.9 years from three different regions in Vietnam including rural and urban areas, which found that 11.3% of children had anaemia [34]. This apparent reduction in the prevalence of anaemia in school children in Vietnam may be due to the implementation of the Improving Children’s Nutrition Status Project, previously known as the National Target Program for Protein Malnutrition Control, in the last 30 years. The Improving Children’s Nutrition Status Project includes activities for anaemia control for children such as micronutrient supplementation and fortification, biannual mass vitamin A supplementation, nutrition education and communication, deworming, hygiene education, and improved sanitation [37].

Despite this possible reduction, anaemia remains a public health issue in school children in Vietnam [8]. As anaemia in childhood has been associated with poor health status, physical activity and school performance [1,3,4,5], efforts are required to reduce its prevalence in this population. The WHO assumes that iron deficiency is responsible for approximately half of all anaemia cases [8] with other contributing factors being chronic inflammatory disorders, infections [6], genetic haemoglobin disorders and co-existing micronutrient deficiencies [1,7]. Due to funding constraints, we were not able to explore the contribution of iron deficiency or other factors to anaemia in our study. However, 57% of children with anaemia in our study had microcytic anaemia, which could suggest iron deficiency, chronic disease, and/or haemoglobinopathies [4]. The other 43% of anaemic children had normocytic anaemia, which could indicate chronic inflammatory conditions [4]. Therefore, further research is needed to understand factors that may contribute to the development of anaemia in school children in Vietnam with the aim of developing strategies for its prevention and control that are targeted directly at this vulnerable population.

We found a significant association between anaemia (i.e., anaemia and normocytic anaemia) and undernutrition. Anaemia and normocytic anaemia were more likely in underweight and wasted children and those in anthropometric failure while anaemia was also more likely in stunted children compared to children who were not underweight, wasted, were not in anthropometric failure, or were not stunted, respectively. These results are consistent with previous research showing that undernourished children are more likely to be anaemic [38,39]. Undernourished children may be at risk of nutrient deficiencies including protein, iron, vitamin A, vitamin B12, folic acid, or riboflavin [40], which may lead to anaemia. Moreover, anaemia arising from some of these micronutrient deficiencies adversely affects the immune system, increasing the risk of infection [41] and worsening the nutritional status leading to further undernutrition [42]. Clearly, efforts to address anaemia among school children in Vietnam should also include strategies with a specific focus on preventing undernutrition.

In the current study, our initial analysis found a negative association between anaemia (i.e., anaemia and normocytic anaemia) and overweight/obesity in contrast to previous studies, some of which failed to report any association between anaemia and overweight/obesity [43,44,45], whereas others detected a positive association between Hb concentration and BMI [43,44]. To further examine the correlation between Hb concentration and BMI among children in the current study, we performed an additional analysis and also found a significant positive relationship between Hb and BMI-for-age z-score. This indicates that here overweight/obese children may have had a higher Hb concentration compared to normal weight or underweight children for reasons that we were not able to explore. We did not assess dietary intakes or the iron status of the children involved in the current study. However, in our earlier research conducted in four primary schools in Hai Phong City in 2012, overweight/obese children aged 6–10 years had higher iron intakes compared with normal weight children (11.1 (SD 4.8) mg/d vs. 8.4 (SD 3.6) mg/d) [46]. If the children in the current study had similar iron intakes to those reported earlier [46], this may partially explain the lower prevalence of anaemia among overweight/obese children compared to those who were not overweight/obese.

We found no evidence of a relationship between anaemia and sex or age, even though in some [10,39] but not all studies [47,48], younger children and boys have been shown to be at greater risk of anaemia than older children and girls. The narrow age range of our children (6–9 years) compared to the wider age range of the children participating in the earlier study (6–19 years) [39] may explain our results.

Anaemia has been shown previously to negatively correlate with socio-economic status, i.e., household wealth and maternal education [4,8]. In contrast, we found no association between anaemia and any of the selected socio-economic factors investigated such as maternal employment, maternal education, or household income, findings in accordance with Alelign et al’s study in Ethiopian children [49]. In our additional analyses, we also found no association between Hb and selected socio-economic factors, in contrast to Amarasinghe et al. who reported a significant positive relationship between parental education and Hb concentrations of Sri Lankan children [44]. Similarly, although not statistically significant, children of mothers with better education also had higher Hb concentrations in our study. However, the lack of statistical significance in our analysis might have resulted from a small number of mothers who reported their education level, i.e., 678 out of 893 mothers compared to 4412 parents from the study by Amarasinghe et al [44], which might have been insufficient to detect the association. Therefore, further research is necessary to understand the relationship between anaemia and socio-economic conditions in Vietnam, which may include other socio-economic factors not investigated in this study.

In summary, anaemia remains a public health problem in Hai Phong City, Vietnam. Children who were underweight, wasted or in anthropometric failure were more likely to be anaemic or have normocytic anaemia compared to children who were not underweight, wasted or in anthropometric failure, respectively. Stunted children were more likely to be anaemic. Overweight/obese children were less likely to be anaemic or have normocytic anaemia. No anthropometric status indicators were associated with the risk of microcytic anaemia. We detected no association between age, sex, maternal employment, maternal education, or monthly household income and anaemia or any of its subtypes. Clearly, future approaches to prevent and control anaemia in Vietnam should target undernourished primary school children and include detailed epidemiological studies to determine the causes of anaemia in this population.

## Figures and Tables

**Table 1:** Characteristics of the study participants.

		N	%
	All Children	893	
Sex	Girls	450	50.4
	Boys	443	49.6
Age (months)	69 to <81	303	33.9
	81 to <92	294	32.9
	92 to ≤108	296	33.2
Stunting ^a^	Not stunted	841	94.7
	Stunted	47	5.3
Underweight ^b^	Not underweight	812	91.2
	Underweight	78	8.8
Wasting ^c^	Not wasted	833	93.8
	Wasted	55	6.2
Overweight/ Obesity ^d^	Not overweight/obese	722	81.3
	Overweight/obese	166	18.7
CIAF ^e^	No anthropometric failure	772	86.7
	Anthropometric failure ^e^	118	13.3
	Wasting only	28	3.2
	Wasting + underweight	23	2.6
	Wasting + stunting + underweight	4	0.4
	Stunting + underweight	31	3.5
	Stunting only	12	1.3
	Underweight only	20	2.3
Maternal employment status	Full time employment	424	63.0
	Self-employed	117	17.4
	Farmer	75	11.1
	Unemployed	57	8.5
Maternal education	Above high school	178	26.3
	High school	229	33.8
	Below high school	271	39.9
Monthly household income (million VND)	<6	199	31.1
	6 to <10	209	32.7
	≥10	232	36.2

a Defined as height-for-age z-score < −2 [32]; b Defined as weight-for-age z-score < −2 [32]; c Defined as BMI-for-age z-score < −2 [32]; d Defined as BMI-for-age z-score > 1 [32]; e Composite Index of Anthropometric Failure (CIAF): height-for-age, weight-for-age, and/or BMI-for-age z-score < −2 [33].

**Table 2:** Prevalence of anaemia (Hb < 115 g/L) and associated demographic, socio-economic and anthropometric factors in 6–9-year-old children in rural areas of Vietnam.

		Total (N)	Anaemia (N)	Prevalence, % (95% CI)	Univariate Analysis ^a^		
					OR (95% CI)	*P*-Value	Global *P*-Value ^b^
All children		893	138	12.9 (8.1, 19.9)			
Sex	Girls (reference)	450	66	12.4 (7.5, 19.8)	1		
	Boys	443	72	13.3 (8.2, 21.1)	1.09 (0.75, 1.58)	0.664	
Age (months)	69 to <81 (reference)	303	54	15.4 (9.3, 24.5)	1		0.158
	81 to <92	294	45	12.9 (7.6, 21.0)	0.81 (0.52, 1.27)	0.366	
	92 to ≤108	296	39	10.4 (5.9, 17.5)	0.64 (0.40, 1.00)	0.055	
Maternal employment status	Self-employed (reference)	117	15	11.2 (5.4, 21.7)	1		0.676
	Full time employment	424	67	11.8 (6.6, 20.0)	1.06 (0.57, 1.99)	0.853	
	Farmer	75	13	12.2 (5.6, 24.5)	1.11 (0.47, 2.58)	0.815	
	Unemployed	57	11	17.2 (8.0, 33.2)	1.65 (0.68, 4.02)	0.268	
Maternal education	Above high school (reference)	178	26	13.8 (7.8, 23.2)	1		0.559
	High school	229	33	11.1 (6.0, 19.5)	0.98 (0.54, 1.76)	0.939	
	Below high school	271	47	11.3 (6.0, 20.3)	1.26 (0.72, 2.20)	0.425	
Monthly household income (million VND)	<6 (reference)	199	31	13.0 (7.1, 22.5)	1		0.538
	6 to <10	209	28	10.4 (5.5, 18.6)	0.78 (0.44, 1.37)	0.383	
	≥10	232	39	13.4 (7.5, 22.8)	1.04 (0.61, 1.77)	0.893	
Stunting ^c^	Not stunted (reference)	841	125	12.3 (7.7, 19.2)	1		
	Stunted	47	13	24.5 (12.6, 42.4)	2.31 (1.15, 4.61)	0.018	
Underweight ^d^	Not underweight (reference)	812	117	12.0 (7.5, 18.6)	1		
	Underweight	78	21	23.6 (13.2, 38.4)	2.27 (1.30, 3.97)	0.004	
Wasting ^e^	Not wasted (reference)	833	122	12.1 (7.5, 18.8)	1		
	Wasted	55	16	26.0 (14.0, 43.1)	2.55 (1.35, 4.84)	0.004	
Overweight/ Obesity ^f^	Not overweight/obese (reference)	722	124	14.4 (9.2, 21.7)	1		
	Overweight/obese	166	14	7.9 (4.0,14.8)	0.51 (0.28, 0.92)	0.026	
CIAF ^g^	No anthropometric failure (reference)	772	108	11.6 (7.2, 18.1)	1		
	Anthropometric failure	118	30	22.0 (12.9, 34.9)	2.16 (1.34, 3.48)	0.002	

a Generalised linear mixed models including the covariate as a fixed effect and school as a random effect; b Overall p-value for the association between the outcome and the covariate; c Defined as height-for-age z-score < −2 [32]; d Defined as weight-for-age z-score < −2 [32]; e Defined as BMI-for-age z-score < −2 [32]; f Defined as BMI-for-age z-score > 1 [32]; g Composite Index of Anthropometric Failure: height-for-age, weight-for-age, and/or BMI-for-age z-score < −2 [33].

**Table 3:** Prevalence of microcytic anaemia (Hb < 115 g/L and MCV < 80 fL) and associated demographic, socio-economic and anthropometric factors in 6–9-year-old children in rural areas of Vietnam.

		Total (N)	Microcytic Anaemia (N)	Prevalence, % (95% CI)	Univariate Analysis ^a^		
					OR (95% CI)	*P*-Value	Global *P*-Value ^b^
All children		860	77	7.9 (5.3, 11.6)			
Sex	Girls (reference)	429	32	6.5 (4.0, 10.4)	1		
	Boys	431	45	9.2 (5.9, 14.0)	1.45 (0.90, 2.35)	0.127	
Age (months)	69 to <81 (reference)	272	26	8.2 (4.9, 13.4)	1		0.444
	81 to <92	292	29	9.0 (5.5, 14.3)	1.11 (0.63, 1.96)	0.720	
	92 to ≤108	296	22	6.4 (3.7, 10.8)	0.76 (0.42, 1.39)	0.377	
Maternal employment status	Full time employment (reference)	412	39	4.3 (1.7, 10.5)	1		0.167
	Self-employed	110	5	8.5 (5.5 13.0)	2.06 (0.79, 5.43)	0.142	
	Farmer	72	5	5.8 (2.2, 14.3)	1.37 (0.38, 5.02)	0.632	
	Unemployed	57	8	13.7 (6.6, 26.3)	3.54 (1.09, 11.52)	0.036	
Maternal education	Above high school (reference)	173	15	8.2 (4.7, 14.1)	1		0.986
	High school	221	19	7.8 (4.5, 13.0)	0.94 (0.45, 1.94)	0.865	
	Below high school	262	23	8.0 (4.8, 12.9)	0.97 (0.48, 1.95)	0.929	
Monthly household income (million VND)	<6 (reference)	192	14	7.0 (3.9, 12.1)	1		0.771
	6 to <10	204	19	8.8 (5.3, 14.3)	1.30 (0.63, 2.67)	0.483	
	≥10	224	18	7.6 (4.5, 12.5)	1.10 (0.53, 2.28)	0.797	
Stunting ^c^	Not stunted (reference)	809	70	7.6 (5.1, 11.2)	1		
	Stunted	46	7	13.5 (6.1, 27.5)	1.90 (0.81, 4.45)	0.141	
Underweight ^d^	Not underweight (reference)	783	67	7.5 (5.0, 11.1)	1		
	Underweight	74	10	12.0 (6.0, 22.5)	1.67 (0.81, 3.43)	0.163	
Wasting ^e^	Not wasted (reference)	804	71	7.7 (5.2, 11.4)	1		
	Wasted	51	6	10.6 (4.5, 23.1)	1.42 (0.58, 3.44)	0.448	
Overweight/ obesity ^f^	Not overweight/obese (reference)	695	67	8.5 (5.7, 12.4)	1		
	Overweight/obese	160	10	6.0 (3.0, 11.6)	0.69 (0.34, 1.40)	0.307	
CIAF ^g^	No anthropometric failure (reference)	744	63	7.4 (4.9, 11.1)	1		
	Anthropometric failure	113	14	11.0 (6.0, 19.3)	1.54 (0.82, 2.86)	0.178	

a Generalised linear mixed models including the covariate as a fixed effect and school as a random effect; b Overall p-value for the association between the outcome and the covariate; c Defined as height-for-age z-score < −2 [32]; d Defined as weight-for-age z-score < −2 [32]; e Defined as BMI-for-age z-score < −2 [32]; f Defined as BMI-for-age z-score > 1 [32]; g Composite Index of Anthropometric Failure: height-for-age, weight-for-age, and/or BMI-for-age z-score < −2 [33].

**Table 4:** Prevalence of normocytic anaemia (Hb < 115 g/L and MCV 80–90 fL) and associated demographic, socio-economic and anthropometric factors in 6–9-year-old children in rural areas of Vietnam.

		Total (N)	Normocytic Anaemia (N)	Prevalence, % (95% CI)	Univariate Analysis ^a^		
					OR (95% CI)	*P*-Value	Global *P*-Value ^b^
All children		860	59	5.3 (2.9, 9.5)			
Sex	Girls (reference)	429	32	5.9 (3.1, 11.0)	1		
	Boys	431	27	4.7 (2.4, 9.0)	0.78 (0.46, 1.34)	0.373	
Age (months)	69 to <81 (reference)	272	26	7.5 (3.9, 14.1)	1		0.102
	81 to <92	292	16	4.2 (2.0, 8.7)	0.54 (0.28, 1.04)	0.069	
	92 to ≤108	296	17	4.4 (2.1, 8.9)	0.56 (0.29, 1.07)	0.077	
Maternal employment status	Full time employment (reference)	412	27	4.9 (1.4, 16.3)	1		0.477
	Self-employed	110	9	3.1 (0.9, 9.9)	0.62 (0.27, 1.41)	0.258	
	Farmer	72	8	5.4 (1.4, 18.5)	1.10 (0.38, 3.16)	0.866	
	Unemployed	57	3	3.1 (0.6, 14.1)	0.62 (0.15, 2.47)	0.495	
Maternal education	Above high school (reference)	173	11	3.0 (0.8, 10.2)	1		0.309
	High school	221	13	3.0 (0.9, 9.9)	1.01 (0.42, 2.41)	0.989	
	Below high school	262	23	4.8 (1.5, 14.4)	1.64 (0.73, 3.36)	0.230	
Monthly household income(million VND)	<6 (reference)	192	16	4.6 (1.4, 14.5)	1		0.126
	6 to <10	204	9	2.1 (0.6, 7.6)	0.45 (0.19, 1.06)	0.066	
	≥10	224	20	4.5 (1.3, 14.0)	0.97 (0.48, 1.99)	0.942	
Stunting ^c^	Not stunted (reference)	809	54	5.1 (2.8, 9.3)	1		
	Stunted	46	5	9.0 (3.2, 22.9)	1.82 (0.68, 4.90)	0.236	
Underweight ^d^	Not underweight (reference)	783	48	4.7 (2.6, 8.6)	1		
	Underweight	74	11	12.0 (5.4, 24.3)	2.74 (1.33, 5.64)	0.006	
Wasting ^e^	Not wasted (reference)	804	49	4.7 (2.6, 8.5)	1		
	Wasted	51	10	15.7 (7.0, 31.4)	3.76 (1.73, 8.17)	0.001	
Overweight/ obesity ^f^	Not overweight/obese (reference)	695	55	6.2 (3.5, 10.9)	1		
	Overweight/obese	160	4	2.2 (0.7, 6.4)	0.33 (0.12, 0.94)	0.038	
CIAF ^g^	No anthropometric failure (reference)	744	44	4.6 (2.5, 8.3)	1		
	Anthropometric failure	113	15	10.5 (5.1, 20.5)	2.45 (1.30, 4.64)	0.006	

a Generalised linear mixed models including the covariate as a fixed effect and school as a random effect; b Overall p-value for the association between the outcome and the covariate; c Defined as height-for-age z-score < −2 [32]; d Defined as weight-for-age z-score < −2 [32]; e Defined as BMI-for-age z-score < −2 [32]; f Defined as BMI-for-age z-score > 1 [32]; g Composite Index of Anthropometric Failure: height-for-age, weight-for-age, and/or BMI-for-age z-score < −2 [33].

**Table 5:** Intracluster correlation coefficient (ICC) estimates for each type of anaemia ^a^.

	ICC (95% CI)
Anaemia ^b^	0.146 (0.088, 0.204)
Microcytic anaemia ^c^	0.085 (0.053, 0.116)
Normocytic anaemia ^d^	0.065 (0.034, 0.096)

a ICC estimated under a generalised linear mixed model with school as a random effect and no covariates; b Hb < 115 g/L; c Hb < 115 g/L and MCV < 80 fL; d Hb < 115 g/L and MCV 80–90 fL.
